# One Health – a strategy for resilience in a changing arctic

**DOI:** 10.3402/ijch.v74.27913

**Published:** 2015-09-01

**Authors:** Bruce A. Ruscio, Michael Brubaker, Joshua Glasser, Will Hueston, Thomas W. Hennessy

**Affiliations:** 1 Office of International Health and Biodefense, Bureau of Oceans and International Environmental and Scientific Affairs, U.S. Department of State, Washington, DC, USA; 2 Center for Climate and Health, Alaska Native Tribal Health Consortium, Anchorage, AK, USA; 3 Global Leadership Programs, Center for Animal Health and Food Safety, College of Veterinary Medicine, University of Minnesota, St Paul, MN, USA; 4 Arctic Investigation Program, Division of Preparedness and Emerging Infections, National Center for Emerging and Zoonotic Diseases, Centers for Disease Control and Prevention (CDC), Atlanta, GA, USA

**Keywords:** Arctic, circumpolar, One Health, infectious disease, climate change, policy, health policy

## Abstract

The circumpolar north is uniquely vulnerable to the health impacts of climate change. While international Arctic collaboration on health has enhanced partnerships and advanced the health of inhabitants, significant challenges lie ahead. One Health is an approach that considers the connections between the environment, plant, animal and human health. Understanding this is increasingly critical in assessing the impact of global climate change on the health of Arctic inhabitants. The effects of climate change are complex and difficult to predict with certainty. Health risks include changes in the distribution of infectious disease, expansion of zoonotic diseases and vectors, changing migration patterns, impacts on food security and changes in water availability and quality, among others. A regional network of diverse stakeholder and transdisciplinary specialists from circumpolar nations and Indigenous groups can advance the understanding of complex climate-driven health risks and provide community-based strategies for early identification, prevention and adaption of health risks in human, animals and environment. We propose a regional One Health approach for assessing interactions at the Arctic human–animal–environment interface to enhance the understanding of, and response to, the complexities of climate change on the health of the Arctic inhabitants.

While circumpolar collaboration on health and the environment has never been greater, the magnitude and complexity of the health challenges facing the Arctic are daunting. Looking forward, a comprehensive approach to health will catalyse actions that protect the health of the region's people, animals and environment. This can be achieved with a regional One Health approach among the nations and permanent participants of the circumpolar north. Understanding the health risks of climate change in the Arctic will require scientists, policy makers, communities and public health experts to collaborate beyond the confines of their disciplines and borders, and One Health provides an approach to detect the emergence of climate-sensitive health threats in the region. As a shared regional approach, One Health can enhance disease prevention and resiliency for Arctic inhabitants.

## The grand challenge of Arctic health

While biologists, climatologists, geographers and oceanographers define the Arctic differently, for the purpose of this article, the circumpolar region consists of 27 regions wholly or partly located above 60°N and includes approximately 44 million square kilometres. The countries of this region include Canada, the Kingdom of Denmark (specifically, Greenland and the Faroe Islands), Finland, Iceland, Norway, Russia, Sweden and the United States. The region is home to diverse environments and populations of plants, animals and people living in some of the most extreme conditions on the planet. The physical and biological environments are diverse and include temperate rainforest, boreal forest, tundra, polar desert and cold oceans. There are approximately 10 million human inhabitants in the region that are ethnically diverse with dozens of Indigenous groups (1). Many of these people still have traditional subsistence economies based upon gathering wild plants, hunting fishing and herding of reindeer (2, 3).

The region is known as being both rugged and resilient, due in part to the persistent cold temperatures and the largely frozen condition of the land and sea. However, as the Arctic warms and the lands and ice thaw, the region is increasingly fragile. Arctic temperatures have risen at twice the rate of other parts of the world resulting in decreased sea ice, coastal erosion, changes in precipitation magnitude and frequency, permafrost thawing and altered distribution of plant and animal species (4). The associated health risks for humans and animals include potential changes in pathogen and vector demographics affecting disease patterns; degradation of drinking water quality and availability, food quality and availability, and changes in animal and plant species health, among others (5–7). Rapid change and recognition of the emerging health threats have resulted in a concerted effort to enhance regional and international partnerships to share best practices in disease surveillance and prevention strategies (8, 9). Understanding the evolving health threats and anticipating and managing risks influenced by the dynamic impacts of climate change in the Arctic will require innovative science, novel tools and even greater integration of efforts. The implications of health risks – to Arctic populations and those beyond – calls for broad and diverse stakeholder collaborations to advance the fundamental understanding of emerging health threats, and the development of shared initiatives that decrease vulnerabilities of human and animal communities and the environment. An integrated and holistic approach will be essential for providing the evidence of links between climate change and health risks to support sound policy development.

## One Health

One Health represents an approach for developing and sustaining broad transdisciplinary collaboration for the early identification, prevention and mitigation of health risks in human, animals and the environment. While there are slightly varying definitions of One Health, most are similar to this European Union definition:

One Health is an integrated approach to health that focuses on the interactions between animals, humans and their diverse environments. It encourages collaborations, synergies and cross-fertilization of all professional sectors and actors in general whose activities may have an impact on health. (10)

One Health recognizes that understanding these interactions and interdependencies necessitate an integrated perspective (11, 12).

One Health is not new, though it has gained significant attention over the past decade. An integrated approach to animal, human and environmental health issues can be traced to ancient times. The concept of One Health in the modern age evolved from the theory of One Medicine developed by Sir William Osler in the late 1800s and further elaborated by Calvin Schwabe in the 1970s (13, 14). Recent attention to One Health can, in part, be attributed to acknowledgement of complex health-related issues associated with rapidly growing populations, increasing speed and magnitude of human travel and migrations, environmental degradation, and disturbance, societal instability and climate change. Visible effects of these forces are expansion, range shift and new emergence of animal, plant and human diseases (15, 16). The vast majority of emerging disease outbreaks over the past 30 years have been due to zoonotic or vector-borne disease. These health risks are evident in the emergence, re-emergence and/or global spread over the past decade of a wide range of infectious diseases: Hanta virus, Ebola, H1N1 influenza (which reached pandemic levels in 2009), Highly Pathogenic Avian Influenza (H5N1), West Nile virus, Rift Valley Fever virus, norovirus, Dengue and Chikungunya viruses, severe acute respiratory syndrome, Marburg, *E. coli* O157:H7, *Yersinia pestis* (Plague) and *Bacillus anthracis* (anthrax) (17). While animal health, human health and environmental health are intricately linked, our approach to understand these health risks are mostly independent. The needed collaboration and communication across and between scientific disciplines has been lacking or non-existent. One Health advances a sustained partnership across disciplines and has demonstrated accomplishments in understanding complex health risks (see www.onehealthinitiative.com/ and www.cdc.gov/onehealth/in-action/index.html). Further, by focusing on the interface of humans, animal and the environment, One Health can help predict outbreaks of disease through a more in-depth understanding of the development and transmission of diseases.

## One Health concept in the Arctic

There is a need to advance the fundamental understanding of climate change impacts on Arctic health and provide the quantitative evidence base for enhanced decision-making that will lead to scientifically sound and societally supported public policies. One Health is a particularly well-suited approach to advance the understanding of the constantly changing health threats resulting from the direct and indirect impacts of climate change in the Arctic. Specifically, an Arctic One Health approach can enhance surveillance capacity to monitor climate-sensitive health risks; advance a regional baseline understanding of the interaction between human and animal disease and disease vectors and increase the understanding of the relationship between climate changes and emerging of health risks and benefits.

The circumpolar north provides an optimal venue for a regional One Health approach. First, the components of a One Health approach are already evident (18). There is a strong history of local, national, regional and international cooperation among diverse stakeholders in addressing human, animal and environmental health issues (19, 20). Second, One Health can enhance the exchange of information and take into account local and traditional knowledge and participatory community-based approaches in identifying and responding to health issues. At the core of a One Health approach are those stakeholders in close proximity to the natural environment and include local communities and indigenous peoples. Third, there are on-going programs, systems and networks working in close collaboration that include local and regional government, multidisciplinary science communities, research institutes, academia, non-governmental agencies, the private sector, civil societies, native organizations and other stakeholders (21, 22). One example of a transdisciplinary organization is the Alaska One Health Group. The One Health Group was formed in 2013 and is hosted by the Alaska Native Tribal Health Consortium and the U.S. Centers for Disease Control, Arctic Investigations Program. The group participants include professionals in the fields of plant, wildlife and environmental health and management, and public health, among others with representative from Canada, Alaska, and other parts of the United States. They meet quarterly to discuss emerging One Health issues, to consider events that are indicative of environmental and climate change and to provide a forum for identifying areas of common interest and collaboration (see www.anthc.org/chs/ces/climate/aohg.cfm). The group maintains a current web accessible One Health Map, which provides a visual aide to help track emerging and trending events. The maps include events screened from news reports, selected posts from the Local Environmental Observer (LEO) Network and additions made by group members. Updates on trending events are also provided and presentations by topic experts. Trending topics have included important food security events such as increases in toxicity and frequency of harmful algal blooms and die-offs of fish, sea mammals and birds.

Fourth, Arctic stakeholders are experienced at integrating collaborative scientific and health policy development across disciplines, cultures and borders (23). Networks are in place that coordinate different aspects of Arctic health including environmental monitoring, animal and human disease surveillance and reporting (9). Fifth, there is recognition of the need for an operational, multidisciplinary and holistic model for assessing and responding to all health risks (18). Finally, there is a track record of policy makers receptive to and influenced by knowledge from diverse scientific and traditional disciplines. For example, scientific data and Indigenous traditional knowledge have resulted in evidence-based policy development in the United States, Canada and Nordic countries on research agendas, interpreting data and local community policy formulation (24).

It is also important to highlight the efforts that have enhanced international partnerships for sharing best practices in disease surveillance and prevention strategies on health risks across the circumpolar countries (22). International collaborations on policies, programmes and initiatives in the Arctic have supported the integration of stakeholders and disciplines since human health became a specific focus for research in 1957 with the establishment of the Nordic Council committee for Arctic Medical Research (25, 26) (see Table I). Two working groups under the Artic Council focussing on human health include the Arctic Monitoring and Assessment Program and the Sustainable Development Working Group formed in 1991 and 1998, respectively (9). In 2010, the Arctic Council established the Arctic Human Health Expert Group (AHHEG) to more fully integrate the assessment of human health risks with environmental issues (27). The Charter of the AHHEG is to advance collaboration between all stakeholders on integrated efforts to attendant human health issues with knowledge gained through environmental and community-based research. In 2011, the Health Ministries of the Arctic States issued the Nuuk Declaration, which describes the prioritized areas of concern and actions on health issues and specifically identifies circumpolar cooperation on assessing climate change impacts on health (28). Also in 2011, the Arctic Council established the International Circumpolar Surveillance Climate Change and Infectious Disease Group to strengthen the integration of animal and human health systems to minimize disease emergence in the Arctic (18, 29, 30).

**Table I:** Summary of Arctic international health policies/initiatives and One Health approach

Arctic International Health Programmes					
	
Health effort/initiative	Year formed	Membership	Mission/objective	Established through	One Health approach
Committee for Arctic Medical Research	1957	Medical/Academic representative from Denmark, Finland, Iceland, Norway and Sweden	Advise Nordic Council on medical research in the Arctic	Nordic Council	Regional collaboration in assessing human health risks
Nordic Council for Arctic Medical Research	1966	Medical Officers and Academics from Denmark, Finland, Iceland, Norway and Sweden	Promote Arctic medical research in the Nordic Countries	Committee for Arctic Medical Research	Focus on unique health problems susceptible to multidiscipline solutions across national boundaries of the Arctic
International Union for Circumpolar Health	1981	The American Society for Circumpolar Health, the Nordic Society for Arctic Medical Research, the Siberian Branch of the Russian Academy of Science, Canadian Society for Circumpolar Health The Medical Section and the Danish/Greenlandic Society of Circumpolar Health	Contribute to the body of scientific, medical and public health research data for the circumpolar regions and globally. Promotes circumpolar collaboration and co-operation in health and medicine	International Circumpolar Health Symposium	Encourages research and exchange of scientific information across circumpolar health sciences disciplines and promotes participation of Indigenous peoples
International Arctic Science Committee, Social and Human Work Group	1990	Representatives from Arctic Council, International Council for Science, World Climate Research Program, Scientific Committee on Antarctic Research, International Permafrost Association, Pacific Arctic Group, International Arctic Social Sciences Association, Association of Polar Early Career Scientists and International Association of Cryospheric Sciences	Initiate, develop and coordinate scientific activity in the Arctic region. Provide objective and independent scientific advice to the Arctic Council and other organizations on issues of science affecting the management of the Arctic region	International Council of Scientific Unions	Facilitates and promotes multidisciplinary research for a greater scientific understanding of the Arctic region issues
Arctic Council's Arctic Monitoring and Assessment Program, Sustainable Development Working Group, Arctic Contaminants Action Program, Conservation of Arctic Flora and Fauna, Emergency Prevention, Preparedness and Response, Protection of the Arctic Marine Environment	1996	US, Canada, Kingdom of Denmark, Iceland, Norway, Sweden, Finland, the Russian Federation, Indigenous communities	Monitoring and assessing living conditions of Arctic residents, including health	The Arctic Council	Working groups of multidisciplined experts, permanent participants and other stakeholders advancing cooperation and coordination on critical Arctic issues, including the environment, and animal and human health
The Northern Dimension (ND) Partnership in Health and Social Well Being	2003	Membership of Canada, Denmark, Estonia, Finland, France, Germany, Iceland, Latvia, Lithuania, Poland, Russia, Sweden and 8 affiliated organizations	Promote sustainable development in the ND area by improving human health and social well-being through co-operation	The Oslo Declaration	Multidisciplined cooperation to prevention of communicable diseases and lifestyle-related diseases
Joint Working Group on Health and Related Social Issues Of the Joint Barents Euro-Arctic Council – Barents Regional Council working groups	2003	Members from national and local regions, including Iceland, Norway, Finland, Sweden, the north-western Russian Federation and the Kingdom of Denmark	Focus on communicable disease prevention, health promotion and access to primary care and social services	Barents Euro-Arctic Council	Multidisciplined stakeholder effort on prevention and response to communicable diseases; lifestyle-related health and social issues
International Network for Circumpolar Health Research	2005	A voluntary network of researchers and supporters of researchers based in academic research centres, Indigenous people's organizations, regional health authorities, scientific associations and government agencies	Improve the health of the residents of the circumpolar regions through international cooperation in scientific research	Circumpolar Health Researchers	Broad group of stakeholders, researchers, Indigenous people's organizations, health authorities, scientific associations and government agencies, addressing health of the circumpolar regions through international cooperation
The Arctic Human Health Expert Group (AHHEG)	2010	The AHHEG comprised a range of specialized circumpolar human health professionals	Assist the Arctic Council in better coordinating its human health activities through ecosystem and community-based research. Facilitate collaboration and synergies between all stakeholders in the development of sustainable and integrated approaches to address attendant human health issues	Norwegian Arctic Council Chairmanship	Interdisciplinary group of health experts providing an Arctic region perspective and insight on the relationship between human health and society
Arctic Health Declaration	2011	Representatives of the Permanent Participants of Arctic Council	A framework that guides international cooperation for research and development of Arctic Health	The Arctic Council Nuuk Declaration	International agreement on co-operation in Arctic Health specifically identifying climate change and environmental impacts on circumpolar health
Circumpolar Health Research Network	2012	Formed with the union of the International Network for Circumpolar Health Research (INCHR) and the International Association of Circumpolar Health Publishers (IACHP)	Promote cooperation and collaboration among health researchers engaged in research in the circumpolar region. Facilitate the exchange, communication and dissemination of research results and other health data	INCHR and IACHP	Cooperation and collaboration among multidisciplined health researchers engaged in research in the circumpolar region

## Regionalizing an Arctic One Health approach

Harmonizing existing efforts and creating a sustained regional One Health approach will entail an implementation strategy, supporting policies, a critical mass of engaged stakeholders at both grassroots and leadership levels, and sustained commitment in the form of funding and time. Paradigm shifts are not easy. Assessments of the adoption of the One Health approach have concluded that while the concept and principles have been broadly accepted and endorsed, operationalizing has been more challenging (17, 31). These challenges include poorly defined unifying One Health efforts, a lack of One Health champions and partners, limited resource and policy hurdles, among others. However, calls for guidance on how to move beyond concept has resulted in recommendations and roadmaps outlining steps, programme metrics and programme assessments to operationalize One Health (32, 33).

An implementing strategy for Arctic One Health approach will benefit from using one of the recently developed One Health operationalizing “road maps.” Two examples include the work done by the University of Minnesota and US Department of Agriculture (USDA), and Andrea Meisser and Anne Levy Goldblum (34, 35). Developing a strategy with process steps, progress measures and well-defined milestones will be crucial in obtaining broad-based support for a regional One Health effort. The implementation strategy process can, and should, assess vulnerabilities, evaluate alternative strategies and programmes for health risks identification and assessments, assess the costs and benefits of those various options and promote their adoption and/or adaptation. Tools for moving this strategy forward are described below.

**Fig. 1:**
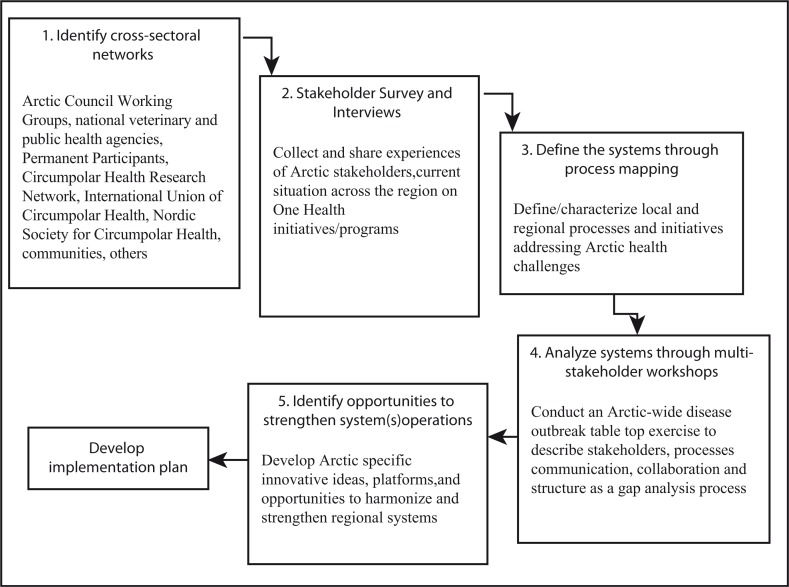
Arctic One Health process mapping. Adapted from *One Health Systems Mapping and Analysis Resource Tool Kit*.

The University of Minnesota and the USDA developed The One Health Systems Mapping and Analysis Resource Toolkit (OH-SMART), an interactive mapping process and framework for a One Health approach to infectious disease threats. The OH-SMART has been successfully used to analyse connections between and among public health, animal health and wildlife sectors, and facilitate improvements in the context of One Health operationalization (36). The tool provides an approach for developing system-based maps detailing agency and stakeholder interactions specific to One Health challenges. The information and data promote stakeholder awareness to analyse processes and strengthen interactions. The OH-SMART process would be applied to increase the awareness of Arctic cross-disciplined partners and activities, to analyse current practices and to create a shared understanding of the current status of One Health approaches. The resulting assessment and baseline information would be used to illuminate the way forward for a One Health approach for the Arctic region, see Fig. 1.

The second tool is an outcome of a 2011 meeting of One Health experts in Bellagio, Italy, to assess the global progress of the adoption of the One Health approach (33). This group of experts conducted a global inventory of 71 on-going One Health efforts and evaluated each programme against an assessment tool for achieving transformational change. While initially developed as an assessment instrument, the authors identified applicability as a change model tool for the transformation of One Health approaches to operationalized programmes.

Five components of change were identified and characterized in this transformation roadmap: Mobilize Commitment, Shared Vision, Organization and Human Resource Alignment, Operationalization and Transformation. Each component involves processes needed to successfully achieve programme transformation. The tool identifies both activities and policies requirements. Further, as a guide with assessment criteria, the road map can be used to measure progress and help identify technical, agencies and policy hurdles and opportunities to achieve an operationalized One Health end point. The transformation would not necessarily proceed in a linear manner and would build on existing programs and activities, incrementally advancing to a fully integrated regional One Health approach (see Table II).

**Table II:** One Health operationalization transformation phases with assessment questions

Process steps for a regional Arctic One Health		

Components	Type of change	Assessment and roadmap
Mobilize Commitment	Perspective and Commitment	- Are all relevant stakeholders identified and engaged? - Do programme participants recognize the need for a multidiscipline, holistic model to address complex Arctic health and climate change? - Do agencies and communities, including permanent participants reflect professional, disciplines, agencies, and organizations working an integrated/collaborative way? - Have relevant policies been reviewed and assessed?
A Shared Vision	Planning and Communication	- Is there an agreed upon definition of One Health for the Arctic? - Is there a shared vision for One Health across all stakeholders - Is there a harmonized “future State” for an Arctic Regional One Health approach? - Is the vision based on the perspectives of Arctic stakeholders? - Have gaps between current state and the vision been identified? - Is there sufficient change readiness (champions) capability to drive/lead the change? - Has a strategy and operational plan been agreed upon to address gaps? - Has a communication strategy been developed and implemented to communicate the vision and reinforce new thinking and behaviour among all stakeholders? - Is there evidence of high level of buy in for the vision? - Is there clear ownership for the plan?
Align Organization and People	Organization and People capability	What evidence is there of the following being aligned with One Health vision/strategy: - Relevant institutions and stakeholders engaged - Relevant policy changes made - Sufficient funding in place - Roles, responsibilities, and authorities clarified - Systems and processes established - One Health leadership capabilities developed - Education and capacity building in place
Operationalize	Implement plan and achieve results	- Are operational plans implemented? - Is a monitoring and evaluation process in place? - Is there evidence of collaboration and data sharing? - Are barriers identified and overcome? - Are quick wins planned for and achieved to sustain and build commitment and momentum? - Do quick wins fit with long-term strategy? - Are contributions and achievements recognized? - Is there an effective and efficient use of resources? - Are feedback and lessons learned used for continuous improvement?
Transformation	Sustainability of change and impact	- Are systems and structures embedded at Arctic region and local level to support transformation? - Is there continuous monitoring, assessment and sharing of best practices?
		- Is One Health leadership demonstrated at all levels, across disciplines and countries? - Are there collaborative and effective partnerships at all relevant levels? - Is there evidence of new collaborations, alliances and partnerships operating effectively? - Have resources been leveraged to allow expansion of the One Health effort? - Is there evidence that One Health has been incorporated into the organizational culture? For Example: • One Health success stories circulated • One health self-reinforcement - To what extent: • Has the future state/vision been achieved? • Does is require modification? • Have the goals and objectives been achieved? - What impact has been achieved? - Has the documented impact been shared broadly with health professionals, policy makers, politicians, partners and stakeholders?

## Conclusion

Climate change impacts on the Arctic region are rapid and dramatic. For Arctic inhabitants, with deep cultural connection to the environment, the associated health risks to humans, animals and the environment are increasingly apparent in everyday life. Currently, we lack a full understanding of these risks to humans, animals and the Arctic environment. Now it is the time for a new comprehensive perspective of climate change impacts on Arctic health.

One Health is a transdisciplinary approach ideally suited for addressing health issues in complex systems such as the Arctic. The One Health approach promotes collaborative approaches to the collection, analysis and interpretation of a wide range of data to anticipate and respond to the rapidly changing environment and its health impacts on human and animal communities. A One Health approach can provide critical lead time and early warning of impending dangers while stimulating more innovative collaborative intervention options for prevention and response.

An integrated One Health approach addressing the potential health effects at the human–animal–environment interface will enhance the resilience of Arctic communities and the environment in the circumpolar region. Greater scientific understanding of the threats can contribute new tools for effective policy to reduce the burden of health risks and support capacity-building and preparedness. These tools include methods for assessing vulnerability, health and disease screening strategies and programmes for characterizing climate risks, identifying adaptation options, and weighing the costs and benefits of different policies.

Consideration should be given to regional One Health approach in the Arctic. Recently developed One Health programme assessment instruments and change models can help the multiple affected stakeholders and communities catalyse transformational changes in behaviours, infrastructure and capacity. Additionally, the tools can be used as metrics to assess progress, and to report success and hurdles to stakeholders, the community and policy makers. A regional One Health approach, with multiple disciplines working together locally, nationally and internationally at the human–animal–environment interface will significantly contribute to effectively manage climate change and its impact on the health of the Arctic.

The health impacts of climate change in the Arctic are real and expanding. While adopting a One Health transdisciplinary approach represents a major paradigm shift, many short-term opportunities exist for quick wins despite limited resources. An incremental approach following the innovation maxim “Think big, start small, scale fast” has the potential for significant sustained health benefits for the Arctic into the future.

## Glossary

**Ecohealth** – The health of ecosystems representing the complex interactions between people, social and economic conditions, culture and the natural environment.

**Holistic approach** – A systems approach. A focus on, or concerned with, the complete system rather than with the analysis of, treatment of, or dissection into parts.

**Resiliency** – The ability to absorb perturbations and disturbances before fundamental changes occur in the system (36). The ability to successfully adapt to adversity.

**Transdisciplinary** – Collaboration in which exchanging information, altering discipline-specific approaches, sharing resources and integrating disciplines achieves a common scientific goal (37).
